# Night-time population consistently explains the transmission dynamics of coronavirus disease 2019 in three megacities in Japan

**DOI:** 10.3389/fpubh.2023.1163698

**Published:** 2023-06-21

**Authors:** Yuta Okada, Syudo Yamasaki, Atsushi Nishida, Ryosuke Shibasaki, Hiroshi Nishiura

**Affiliations:** ^1^School of Public Health and Graduate School of Medicine, Kyoto University, Kyoto, Japan; ^2^Research Center for Social Science & Medicine, Tokyo Metropolitan Institute of Medical Science, Tokyo, Japan; ^3^Tokyo Center for Infectious Disease Control and Prevention, Tokyo, Japan; ^4^Center for Spatial Information Science, The University of Tokyo, Tokyo, Japan; ^5^Department of Socio-Cultural and Socio-Physical Environmental Studies, The University of Tokyo, Kashiwa, Japan

**Keywords:** severe acute respiratory syndrome virus 2 (SARS-CoV2), mobility, public health and social measures, *de facto* population, cluster

## Abstract

**Background:**

Mobility data are crucial for understanding the dynamics of coronavirus disease 2019 (COVID-19), but the consistency of the usefulness of these data over time has been questioned. The present study aimed to reveal the relationship between the transmissibility of COVID-19 in Tokyo, Osaka, and Aichi prefectures and the daily night-time population in metropolitan areas belonging to each prefecture.

**Methods:**

In Japan, the *de facto* population estimated from GPS-based location data from mobile phone users is regularly monitored by Ministry of Health, Labor, and Welfare and other health departments. Combined with this data, we conducted a time series linear regression analysis to explore the relationship between daily reported case counts of COVID-19 in Tokyo, Osaka, and Aichi, and night-time *de facto* population in downtown areas estimated from mobile phone location data, from February 2020 to May 2022. As an approximation of the effective reproduction number, the weekly ratio of cases was used. Models using night-time population with lags ranging from 7 to 14 days were tested. In time-varying regression analysis, the night-time population level and the daily change in night-time population level were included as explanatory variables. In the fixed-effect regression analysis, the inclusion of either the night-time population level or daily change, or both, as explanatory variables was tested, and autocorrelation was adjusted by introducing first-order autoregressive error of residuals. In both regression analyses, the lag of night-time population used in best fit models was determined using the information criterion.

**Results:**

In the time-varying regression analysis, night-time population level tended to show positive to neutral effects on COVID-19 transmission, whereas the daily change of night-time population showed neutral to negative effects. The fixed-effect regression analysis revealed that for Tokyo and Osaka, regression models with 8-day-lagged night-time population level and daily change were the best fit, whereas in Aichi, the model using only the 9-day-lagged night-time population level was the best fit using the widely applicable information criterion. For all regions, the best-fit model suggested a positive relationship between night-time population and transmissibility, which was maintained over time.

**Conclusion:**

Our results revealed that, regardless of the period of interest, a positive relationship between night-time population levels and COVID-19 dynamics was observed. The introduction of vaccinations and major outbreaks of Omicron BA. Two subvariants in Japan did not dramatically change the relationship between night-time population and COVID-19 dynamics in three megacities in Japan. Monitoring the night-time population continues to be crucial for understanding and forecasting the short-term future of COVID-19 incidence.

## 1. Introduction

Since the severe acute respiratory syndrome virus 2 (SARS-CoV-2) originated in Wuhan, China (1) and developed into a global pandemic that is still ongoing in many countries, closely monitoring the extent of human-to-human contact at a societal level has been a key issue in public health. Human mobility data in general are widely accepted as one of the most important sources of data for inferring the extent of human-to-human contact, because most contacts outside of households cannot be made without people traveling or staying outside. In fact, several studies have provided evidence on the explanatory power of mobility data and its effectiveness on controlling COVID-19 dynamics (2–5).

Among many data sources on human mobility, the usefulness of mobile phone location data for understanding and predicting coronavirus disease 2019 (COVID-19) dynamics has been revealed by studies in countries with high rates of mobile phone ownership (3, 6, 7). These data are also important for monitoring the effectiveness of non-pharmaceutical interventions by governments (8).

In Japan, several studies have examined the relationship between COVID-19 and mobility data. For example, two studies in Japan on COVID-19 dynamics in 2020 showed a positive relationship between COVID-19 spread and population volumes at several types of locations or time zones, particularly restaurant and bar usage (9, 10). This positive relationship was also observed in other empirical studies in Japan exploring different periods of time or using different sources of mobility data (11, 12).

**Figure 1:**
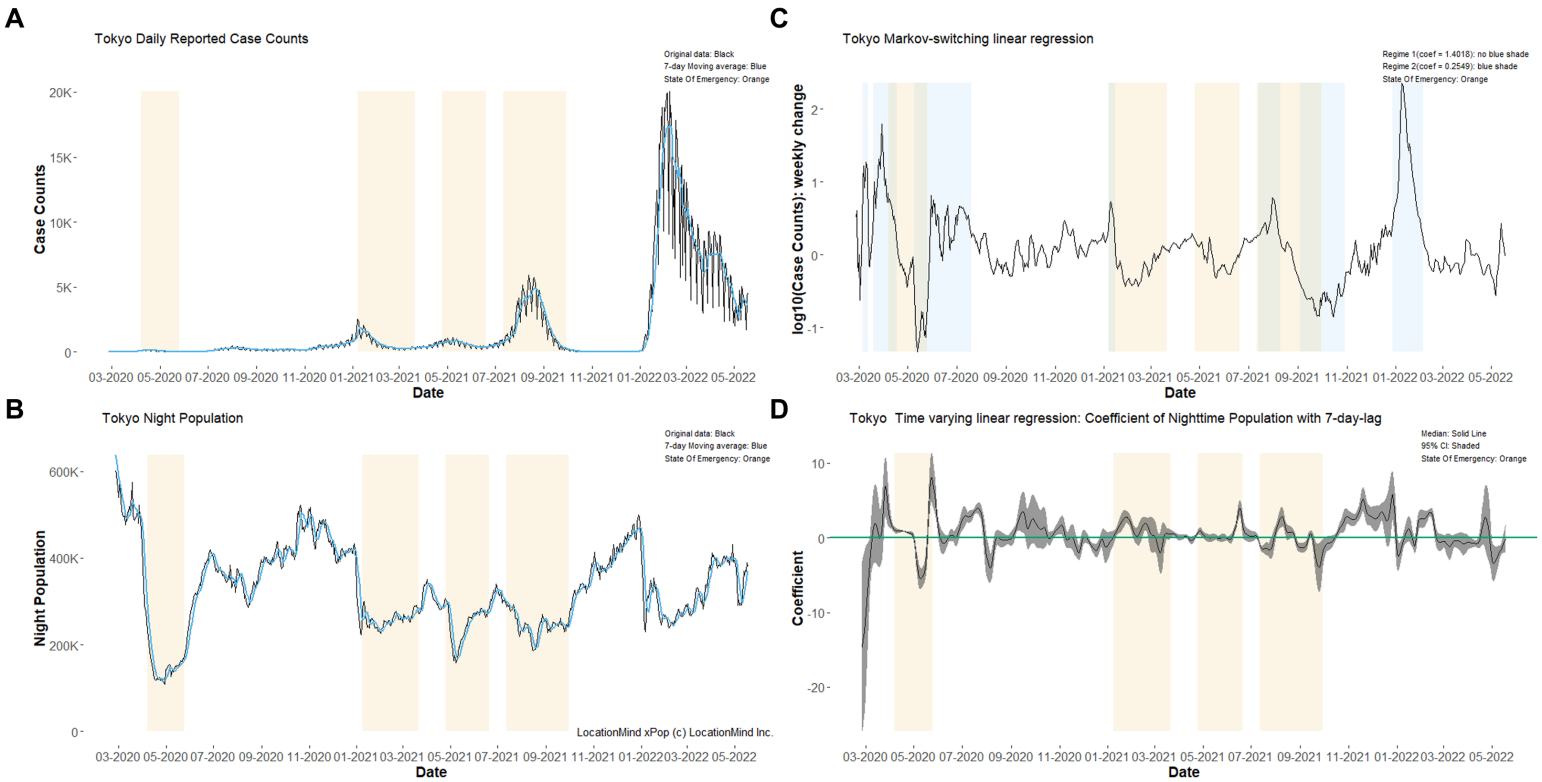
Time series plots showing the daily reported case counts of COVID-19 and daily downtown population from 10:00 PM to 11:59 PM in Tokyo, 2020–22. Tokyo’s **(A)** daily reported COVID-19 case counts and **(B)** Night-time population in designated areas from 10:00 PM to 11:59 PM are shown in the left column (light blue lines show 7-day moving averages). The results of Markov switching linear regressions assuming two hidden states **(C)** and time-varying linear regression **(D)** are shown in the right column. In all figures, light orange shading corresponds to four publicly declared “State of Emergency” periods. For technical details of **(C,D)**, see Supplementary material. The same figures for Aichi and Osaka are shown in the Supplementary material.

Although a positive relationship between mobility and COVID-19 upsurge was observed for a specific period of time in Japan, it remains to be determined whether the effects of social contacts are relatively stable or are highly variable over time, because previous studies in Japan did not analyze data throughout the COVID-19 pandemic from 2020. This issue is particularly important because, if we know in advance about the periods during which the social contact level affects the dynamics of COVID-19, that knowledge could inform how we implement non-pharmaceutical interventions against COVID-19. Moreover, it is possible that the effect of mobility has changed because of vaccinations against COVID-19 or behavioral changes since the emergence of COVID-19. However, to the best of our knowledge, no previous study has tackled the predictive ability of mobility data over a long time-course. It is difficult to correctly estimate the effect throughout a long period of an epidemic, because in the early stages of the pandemic there is no way to know whether the effects are highly time-varying or not. Simply applying linear regressions to explain the dynamics of COVID-19 via mobility data may lead to erroneous results showing high time-dependent variability of the effect of mobility (Tokyo: right column of Figure 1; see Supplementary Figure S1 for Aichi and Osaka), and adequately considering serial correlation in statistical models may cause the results to differ.

One of the key public interests in Japan throughout the COVID-19 pandemic has been on eating and drinking activities that elevate the risk of COVID-19 transmission, and it is widely recognized that such activities are particularly intense in downtown areas at night. Motivated by the insufficiency of evidence on the relationship between night-time drinking or eating activity and COVID-19 dynamics, and the need for such evidence for future rises in COVID-19 caused by novel variants or the emergence of pandemics caused by other pathogens, the objective of the present study was to clarify the relationship between night-time population in the downtown area in three metropolitan areas in Japan and the transmission dynamics of COVID-19. Two linear regression models were employed to appropriately account for the time-dependent relationship between these two datasets.

## 2. Materials and methods

### 2.1. Epidemiological dataset of COVID-19

In Japan, COVID-19 has been designated as a notifiable disease according to The Infectious Disease Control Law, and all confirmed cases are mandatorily reported to the government via local health centers. Confirmatory diagnoses were made either by reverse transcription polymerase chain reaction or rapid diagnostic testing of nose or throat swabs. On the basis of this notification system, daily COVID-19 case count data are openly shared by Japan’s Ministry of Health, Labor, and Welfare, as a function of the reporting date, and we used the open data for the following analyses (13). The dataset shows the daily number of reported COVID-19 cases in each prefecture, created by aggregating the reports from the local health departments in each prefecture. From this dataset, COVID-19 case counts from Tokyo, Osaka, and Aichi were extracted (shared as Supplementary Data).

The day-of-the week-effect observed in the original time series of case counts was intense. To exclude such an effect, as it makes the regression model too complex and less interpretable, for the subsequent analysis we used the 7-day moving average of case counts


$$C_{a}\left(t\right)=\frac{\sum_{i=0}^{6}I_{a}\left(t-i\right)}{7},$$

where $C_{a}\left(t\right)$ is the 7-day moving average of $I_{a}\left(t\right)$, which is the reported case counts on day t in prefecture a. Data from February 26th, 2020, to May 18th, 2022 were used in our study to match the span with the mobility data described below, and to exclude periods with very few COVID-19 case counts.

### 2.2. Mobile phone location data

In the present study, “LocationMind xPop” data on the hourly population volume estimates in selected areas in the Tokyo, Nagoya, and Osaka metropolitan areas were provided by LocationMind Inc. (14). These data were also used in a previous study on COVID-19 and night-time population in Japan (10).

“LocationMind xPop” data refers to people flow data collected by individual location data sent from mobile phones with users’ consent through applications such as “docomo map navi” service (map navi, local guide) provided by NTT DOCOMO, INC. The data are processed collectively and statistically in order to conceal private information. Original location data come from GPS data (latitude, longitude) sent at a frequency of every 5 min at the shortest interval and do not include information that specifies individuals. NTT Docomo, Inc. accounts for about 36.3% of total mobile phone subscribers in Japan (12).

For each metropolitan area, mobile phone trajectories were used to selectively collect population volume that did not involve stay-at-work and stay-at-home behaviors. The areas of interest in this study were selected on the basis of designated areas for monitoring of people flow data by the Cabinet Office (15).

For subsequent analysis, for the same reason as for COVID-19 case counts, we calculated the 7-day moving average of *de facto* night-time population in downtown areas


$$NP_{a}\left(t\right)=\frac{\sum_{i=0}^{6}np_{a}\left(t-i\right)}{7},$$

where $NP_{a}\left(t\right)$ is the 7-day moving average of $np_{a}\left(t\right)$, which is the population staying in the areas of interest between 10:00 PM and 11:59 PM on day t in prefecture a. This particular time of night (i.e., 22:00–00:00) has been specifically used for routine monitoring purposes in Tokyo and for all of Japan on the basis of earlier successful improvements of predictive capability (10).

### 2.3. Variables used in regression analysis

We used the variables mentioned above in natural logarithmic form to ensure equivariance. Our analysis was performed using the 1-week change in $\log\left(C_{a}\left(t\right)\right)$, i.e.,


$$\log\left(C_{a}\left(t\right)\right)-\log\left(C_{a}\left(t-7\right)\right)=\log\left[\frac{C_{a}\left(t\right)}{C_{a}\left(t-7\right)}\right],$$

as a response variable. For explanatory variables, we considered the $\log\left(NP_{a}\left(t\right)\right)$ as well as the daily difference of $\log\left(NP_{a}\left(t\right)\right)$, i.e.,


$$\Delta \log\left(NP_{a}\left(t\right)\right)=\log\left(NP_{a}\left(t\right)\right)-\log\left(NP_{a}\left(t-1\right)\right).$$

For all locations, all three variables tested negative for unit roots using the augmented Dickey–Fuller test using R package CADFtest, to ensure that the whole series of each dataset was valid for regression analysis.

### 2.4. Time-varying regression analysis

First, the following state space model with exogenous variables was applied to conduct time-varying regression analysis, where L was the lag to be determined by exploring the best-fit model:


(1) $$\log\left[\frac{C_{a}\left(t\right)}{C_{a}\left(t-7\right)}\right]=S\left(t\right)+\varepsilon \left(t\right),\varepsilon \left(t\right)\sim N\left(0,\sigma_{\varepsilon }{\;}^{2}\right),$$

(2) $$\begin{array}{c} S\left(t\right)=\beta_{0}\left(t\right)+\beta_{1}\left(t\right)\log\left(NP_{a}\left(t-L\right)\right)\\ +\beta_{2}\left(t\right)\Delta \log\left(NP_{a}\left(t-L\right)\right), \end{array}$$

(3) $$\left[\begin{array}{c} \beta_{0}\left(t\right)\\ \beta_{1}\left(t\right)\\ \beta_{2}\left(t\right) \end{array}\right]=\left[\begin{array}{c} \beta_{0}\left(t-1\right)\\ \beta_{1}\left(t-1\right)\\ \beta_{2}\left(t-1\right) \end{array}\right]+\left[\begin{array}{c} \xi_{0}\left(t\right)\\ \xi_{1}\left(t\right)\\ \xi_{2}\left(t\right) \end{array}\right],$$

(4) $$\left[\begin{array}{c} \xi_{0}\left(t\right)\\ \xi_{1}\left(t\right)\\ \xi_{2}\left(t\right) \end{array}\right]\sim MVN\left(\left[\begin{array}{c} 0\\ 0\\ 0 \end{array}\;\right],\Sigma \left(t\right)\right),$$

(5) σ=7,8,…,14(days)

Eq. (1) is the observation process with state S(t) and observation error ε(t), which is assumed to follow a normal distribution with mean 0 and standard deviation $\sigma_{\varepsilon }$. Eq. (2) is the state equation consisting of time-varying level $\beta_{0}\left(t\right)$ and exogenous variables $\log\left(NP_{a}\left(t-L\right)\right)$ and $\Delta \log\left(NP_{a}\left(t-L\right)\right)$ with time-varying coefficients $\beta_{1}\left(t\right)$ and $\beta_{2}\left(t\right)$, respectively. Intercepts and coefficients $\beta_{i}\left(t\right),i=0,1,2$, are modeled to follow the time-varying process as described in (3) and (4), where Σ is the variance–covariance matrix. The lag ranging from 7 to 14 days was specifically examined, because the mean time delay from infection to reporting was estimated at 13 days during the first wave of the pandemic from March to May 2020, and was then shortened to 11 days from June 2020 and 9 days when the Omicron variant (B.1.1.529) began to spread from January 2022 (16–18) [also see Supplementary material for symptom onset to reporting on the basis of publicly available data from the website managed by the Tokyo Metropolitan Government (18)]. Best-fit models (lag L) were selected on the basis of the Akaike information criterion (AIC).

### 2.5. Fixed-effect regression model

On the basis of the time-varying regression results, linear regression analysis by generalized least squares assuming fixed effects of $\log\left(NP_{a}\left(t-L\right)\right)$ and $\Delta \log\left(NP_{a}\left(t-L\right)\right)$ was also conducted throughout the study period. Below is the model description:


(6) $$\begin{array}{l} \log\left[\frac{C_{a}\left(t\right)}{C_{a}\left(t-7\right)}\right]=\beta_{0}+\beta_{1}\log\left(NP_{a}\left(t-L\right)\right)\\ \;+\beta_{2}\Delta \log\left(NP_{a}\left(t-L\right)\right)+\varepsilon \left(t\right), \end{array}$$

(7) ε(t)=ρε(t−1)+ω(t),

(8) $$\omega \left(t\right)\sim N\left(0,\sigma_{\omega }^{2}\right),$$

(9) L=7,8,…,14(days),

where L is the lag to be determined by exploring the best fit model, ε(t) is the autocorrelated error with coefficient ρ, and ω(t) is white noise following the normal distribution with mean 0 and standard deviation $\sigma_{\omega }$. Models including either $\log\left(NP_{a}\left(t-L\right)\right)$ (fixing $\beta_{2}$ to zero) or $\Delta \log\left(NP_{a}\left(t-L\right)\right)\;$(fixing $\beta_{1}$ to zero) or both were tested. The estimation of model parameters was performed via a Bayesian approach employing the Markov Chain Monte Carlo (MCMC) method. We used weakly informative priors (see Supplementary material) and ran five chains and 3,000 iterations with 1,000 warmups each. Convergence was confirmed with trace plots and the potential scale reduction factor (Gelman-Rubin statistics) Rhat as well as traceplots, and the widely applicable information criterion (WAIC) was used for selection of the best fit model.

### 2.6. Statistical analysis

All statistical and numerical analyses were performed using R version 4.1.2 (The R Project for Statistical Computing, Vienna, Austria) and Stan version 2.21.0. R package KFAS (19) was used in the time-varying regression analysis, and the R package brms version 2.18.0 (20) was used for fixed-effect regression analysis with CmdStan 2.30.1 (21).

## 3. Results

### 3.1. Time-varying regression analysis

**Figure 2:**
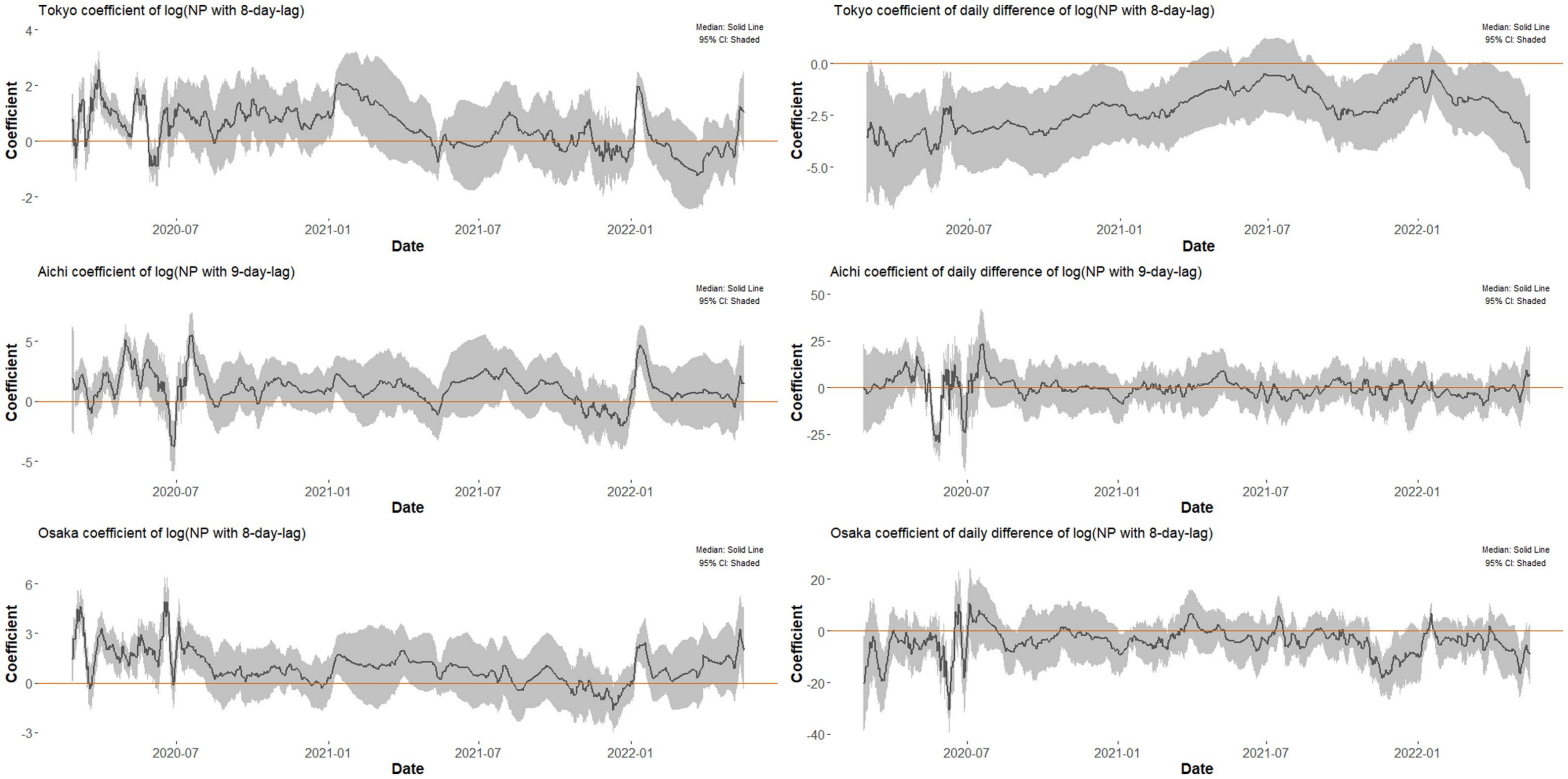
Time series of coefficients of night-time population and its daily change estimated by the time-varying regression models for Tokyo, Aichi, and Osaka, 2020–22. The change of $\beta_{1}\left(t\right)$ and $\beta_{2}\left(t\right)$ over time observed in time-varying regression analysis for Tokyo, Aichi, and Osaka. The shaded areas show the 95% confidence intervals for each estimated value.

For each lag L=7,8,…,14 days, models described in Eqs (1)–(5) were estimated. The lag L of the best-fit model was 8 days in Tokyo, 9 days in Aichi, and 8 days in Osaka (see Supplementary materials for AIC used for best fit model choice). In all locations, the time series of $\beta_{1}\left(t\right)$ and $\beta_{2}\left(t\right)$ are shown in Figure 2. For all locations, the confidence interval of $\beta_{1}\left(t\right)$ stayed above or straddled zero, but in Tokyo and Aichi, there were short periods around mid-2020 when $\beta_{1}\left(t\right)$ turned negative. Regarding $\beta_{2}\left(t\right)$, the confidence interval stayed below zero most of the time throughout the pandemic period. However, the confidence interval of $\beta_{2}\left(t\right)$ in Aichi and Osaka straddled zero most of the time and was sometimes in negative territory. Both $\beta_{1}\left(t\right)$ and $\beta_{2}\left(t\right)$ stayed within a relatively narrow and confined range most of the time.

### 3.2. Fixed-effect regression analysis

For each lag L=7,8,…,14 days, models described in Eqs (6) to (9) are estimated. For each lag L, in addition to models including both $\log\left(NP_{a}\left(t-L\right)\right)$ and $\Delta \log\left(NP_{a}\left(t-L\right)\right)$, models including either one of these variables were also estimated (see Supplementary materials for WAIC used for best fit model choice).

In Table 1, the summary of estimates from best fit models for Tokyo, Aichi, and Osaka are shown.

**Table 1:** Summary of results from best fit models for Tokyo, Aichi, and Osaka, 2020–2022.

	Covariate	Estimate	95% Confidence Interval	
			Lower	Upper
Tokyo	Intercept	−8.676	−12.089	−5.21
	log(NP with 8-day-lag)	0.692	0.427	0.955
	Daily change of log(NP with 8-day-lag)	−2.527	−3.345	−1.713
	First order autoregression coefficient	0.968	0.95	0.986
Aichi	Intercept	−20.165	−27.325	−13.172
	log(Night Population with 9-day-lag)	1.61	1.067	2.168
	First order autoregression coefficient	0.959	0.938	0.979
Osaka	Intercept	−17.167	−28.262	−8.663
	log(NP with 8-day-lag)	1.254	0.638	2.044
	Daily change of log(NP with 8-day-lag)	−3.398	−4.92	−1.843
	First order autoregression coefficient	0.976	0.949	0.997

For Tokyo and Osaka, models with 8-day-lagged night population as well as its daily change were the best fit model, whereas the best fit model for Aichi does not include daily change in night-time population. The estimates for intercept, coefficients of explanatory variables, and the first-order autocorrelation coefficients are shown with 95% confidence intervals.

For Tokyo and Osaka, models with 8-day-lagged night-time population including both $\log\left(NP_{a}\left(t-8\right)\right)$ and $\Delta \log\left(NP_{a}\left(t-8\right)\right)$ were chosen, whereas for Aichi, the model with 9-day-lagged night-time population including only $\log\left(NP_{a}\left(t-9\right)\right)$ was chosen. For every location, the best-fit models suggested a positive correlation between weekly changes in COVID-19 case counts and night-time population with certain lags. For the daily difference in night-time population that was included in the best-fit models for both Tokyo and Osaka, a negative correlation was observed with the weekly change in case numbers. The residuals of fixed-effect models showed that the models were fitted well, but weak autocorrelation and heteroscedasticity in the earlier phases were observed (see Supplementary materials for residuals of these best fit models).

**Figure 3:**
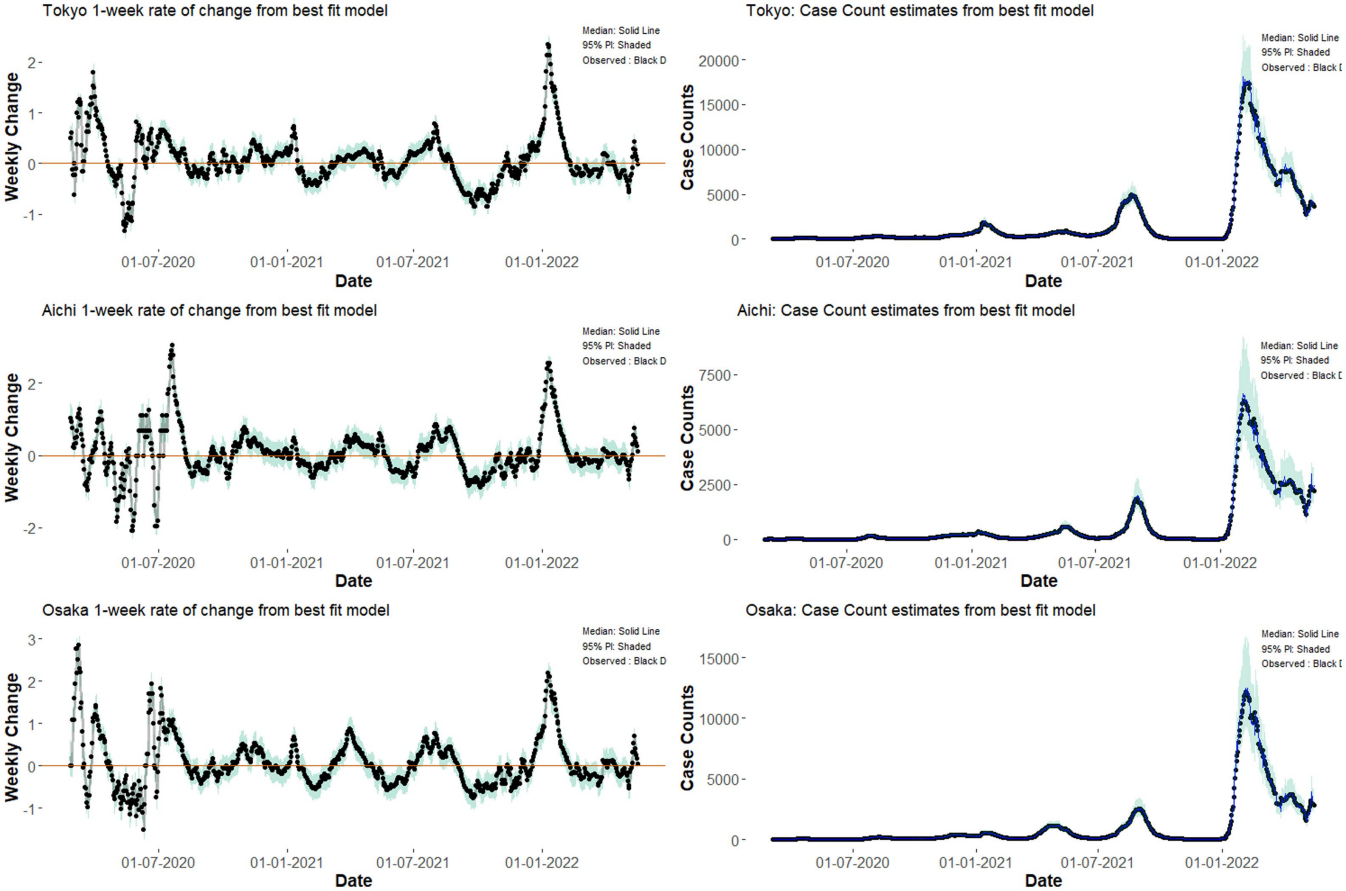
Model estimates of weekly case counts **(left column)** and case counts **(right column)** by the fixed effect regression models for Tokyo, Aichi, and Osaka. Dots represent observed case count by Ministry of Health, Labor, and Welfare, while lines represent the expected value from our fixed-effect regression model. Shaded areas represent the region of 95% prediction intervals computed from the posterior distribution.

Using the fixed-effect model, the values of 1-week rate of changes in COVID-19 counts and the COVID-19 case counts of Tokyo, Aichi, and Osaka are shown in Figure 3, together with model predicted values with 95% prediction intervals. Because the models used in fixed-effect regression consider first-order autocorrelation of residuals, the prediction intervals for each time step are essentially a one-point-ahead forecast with errors.

## 4. Discussion

To the best of our knowledge, the current study is the first in Japan to reveal the long-term relationship between COVID-19 dynamics and night-time downtown populations in metropolitan urban areas where eating and drinking activity are intense. Although it is mechanistically obvious that activities such as drinking or eating indoors are positively linked to the transmission of COVID-19, the time-variability of this link has not been comprehensively explored. The current findings revealed that the effect of night-time population on COVID-19 dynamics was positive to neutral most of the time, and rarely negative over time. The lag considered for night-time population in the best-fit models also appeared to be reasonable, considering that, for major variants of SARS-CoV-2, the mean incubation period of COVID-19 was around 3–7 days (16), and the average lags between symptom onset and reporting in Japan have been estimated to be approximately 3–7 days (17, 18) (also see Supplementary materials). The minor heterogeneity of lags determined through the model fitting process among the three prefectures cannot be explained explicitly based on our result, but it is possible that they root in unknown behavioral differences or differences in the diagnosis-to reporting process of COVID-19 data by local healthcare institutions or governments.

The time-varying regression results revealed that, in addition to the level of night-time population, a neutral to negative correlation was observed between daily difference of night-time population level and COVID-19 transmission for most of the study period. The mechanism underlying this result is not entirely clear. One explanation might be the behavioral changes at the societal level based on expectations of COVID-19 case counts in the near future, or it might also be the reflection of changes in social contact patterns that is characteristic of specific seasons (such as the New Year holiday season). These are no more than guesses, but future research on this topic is of interest.

Fixed-effect regression also revealed that the night-time population level is the key driver of COVID-19 throughout the pandemic period. Although this result is consistent with previous studies in Japan on human mobility and COVID-19 (9–12), the current findings revealed that this positive link between night-time population and COVID-19 transmission was not only limited to a short period but was consistently maintained for a long time over the course of the epidemic. When overviewed as fixed effects, the daily difference in night-time population increased the explainability of best-fit models in Tokyo and Osaka with negative effects. Although this was not true for Aichi, it is possible that, in some locations, the change in night-time population level may account for behavioral changes linked to COVID-19 transmission to some extent, either directly or indirectly.

The current study involved several limitations. The two regression models revealed the correlation between COVID-19 dynamics and explanatory variables such as night-time population and its daily change. It might be possible that any spurious relationship exists between COVID-19 and the explanatory variables included in our models, and that there are other variables not included in our model that may also affect COVID-19 dynamics. About the former issue, generally it is difficult to deny any spurious relationship completely, but due to the definition of lags for variables in our model, there is little chance that the chronological order of possible effects is opposite compared with what we observed in the present study. About variables, several other types of variables are also suggested to affect COVID-19 dynamics. For example, populations staying in other type of locations, residential areas or workplaces, as considered by Nagata et al. (9), were not included in our study. The results of Nagata et al. (9) indicated that the night-time population was the best predictor of COVID-19 dynamics, suggesting that our results may not have been substantially changed by considering other types of locations. However, further analysis considering these locations is warranted if these data are available. The current study also did not include meteorological factors such as temperature and humidity as explanatory variables, which have been suggested to have negative impact on COVID-19 transmission (11, 22) Because these meteorological factors may also exhibit interactions with social contact patterns, we excluded them from consideration. Risk awareness was also considered in previous studies (11) but is difficult to quantify. It is likely that night-time population reflects risk awareness at a societal level to some extent.

The second limitation of the present study is that, in our models, the lags for night-time population were assumed as constants throughout the study period. Not only the substitution of major variants of SARS-CoV-2 but also factors such as the accessibility to hospitals (which might differ among periods or among different epidemic waves) may change the effective lag through which the night-time population affects COVID-19 transmission. It is difficult to adjust for these kinds of changes on the basis of available data when we consider not only known or observable factors but also unmeasured factors. However, the fact that the lag period chosen for best fit models matched that of the fixed-effect regression model in each location suggests that similar underlying correlation structures were maintained throughout the study period. Stepwise regression with sliding windows might also have been an option for our analysis, but we believe that the choice of windows tends to be rather arbitrary, and that temporal dynamics are better elucidated by time-varying regression.

The third limitation is that we only considered three metropolitan areas in Japan using data that focus on social contacts in eating or drinking places. Because our results may not be valid in rural areas in Japan, further studies in other geographical locations are required. Also, unlike the openly accessible mobility data such as Google’s COVID-19 Community Mobility Reports (23), our night-time population data are not open and widely accessible, which might limit validations by other research groups. Nevertheless, the accuracy of our mobility data enabled us to focus almost purely on social contacts at eating or drinking places while excluding residential populations from our scope, which is the key strength of our study. Based on these data, current findings provide important insight for understanding the dynamics of COVID-19 transmission in highly and densely populated areas that represent major parts of the Kanto, Chubu, and Kinki regions of Japan.

Another limitation is that weak autocorrelation was still observed in the residuals of the fixed-effect model even with the inclusion of 1st order autocorrelation structure for residuals. As for autocorrelation, we did not consider higher order autocorrelation considering the nature of COVID-19 transmission that usually occurs within the 1-week scope, but there is room to search candidate variables that might account for the remaining autocorrelation. In addition to autocorrelation, heteroscedasticity was observed mainly in the early phase, which is likely because weekly change rate in the early phase were volatile in all locations due to the relatively small number of COVID-19 cases. Even considering these issues, comparison with the result from time-varying regression suggests that the results from the fixed-effect regression model are also valid.

Despite the limitations mentioned above, we successfully quantified the positive effects of night-time population in downtown areas on COVID-19 transmission using two statistical methods. In addition, we also found that, in Tokyo and Osaka, the daily difference in night-time population may also be a predictor of COVID-19 transmission. Our results emphasize the importance of human mobility data related to eating and drinking activities in society and offer evidence for the effectiveness of public health and social measures targeting high-risk activities or locations for COVID-19 transmission. Specifically, our result shows that, if any dramatic change in population-level immune landscape may occur in the future due to the emergence of SARS-CoV-2 variants with significant immune evasiveness, public health policies should be designed so as to target social contacts that occur in eating or drinking settings including downtown areas. Moreover, this implication from our results can easily be expanded to other pandemics in the future that might be caused by other novel respiratory pathogens including highly pathogenic avian influenza with transmissibility among humans.

## 5. Conclusion

Our results elucidated that night-time population has consistently been a significant predictor of COVID-19 dynamics. The consistency of the effect of night-time population throughout the COVID-19 pandemic up to mid-2022 is especially of note, considering the behavioral changes as well as vaccination campaigns rigorously carried out in Japan.

Even under circumstances where a diminished effort to contain and track COVID-19 paid for by public health officials, our finding encourages close monitoring of mobility indicators particularly focused on places with high levels of eating and drinking activities as a key predictor of surge in COVID-19 cases. Also, from a policy point of view, this finding implicates the importance of targeted financial support for restaurants or pubs that cooperatively close when the endemic situations are bad.

Though our results showed consistent validity throughout the COVID-19 pandemic so far, the validity might change, for example, due to the change in immunity levels at high-risk populations. Thus, future follow-up studies on this topic are warranted.

## Data availability statement

Publicly available datasets were analyzed in this study. This data can be found at: https://covid19.mhlw.go.jp/extensions/public/en/index.html.

## Author contributions

HN conceived the research idea. YO and HN built the statistical models, reviewed the analyses, drafted the manuscript, and approved the final manuscript. YO conducted the statistical analyses on the datasets. All authors gave comments and approved the final version of the manuscript.

## Funding

HN received funding from the Health and Labor Sciences Research Grants (20CA2024, 20HA2007, and 21HB1002), the Japan Agency for Medical Research and Development (JP20fk0108140, JP20fk0108535, and JP21fk0108612), the JSPS KAKENHI (21H03198 and 22K19670), the Environment Research and Technology Development Fund (JPMEERF20S11804) of the Environmental Restoration and Conservation Agency of Japan, the Japan Science and Technology Agency SICORP program (JPMJSC20U3 and JPMJSC2105), and RISTEX program for Science of Science, Technology and Innovation Policy (JPMJRS22B4). AN and HN both received a Health and Labor Sciences grant (21HA2016). AN and SY received financial support from the Tokyo Metropolitan Institute of Medical Science, Japan, and the Infectious Disease Control Department of the Tokyo Metropolitan Government, Japan. The funders had no role in study design, data collection and analysis, decision to publish, or preparation of the manuscript.

## Conflict of interest

The authors declare that the research was conducted in the absence of any commercial or financial relationships that could be construed as a potential conflict of interest.

## Publisher’s note

All claims expressed in this article are solely those of the authors and do not necessarily represent those of their affiliated organizations, or those of the publisher, the editors and the reviewers. Any product that may be evaluated in this article, or claim that may be made by its manufacturer, is not guaranteed or endorsed by the publisher.
