# Supplementary material for: Night-time population consistently explains the transmission dynamics of coronavirus disease 2019 in three megacities in Japan
Source: Front Public Health. 2023 Jun 21;11:1163698. doi: 10.3389/fpubh.2023.1163698 (PMC10321704; doi:10.3389/fpubh.2023.1163698)
Supplement: Supplementary file 1 [file Data_Sheet_1.docx]

Supplementary Material

**Linear regression models used in Figure 1 and Supplementary Figures 1 and 2**

$log\left[ \frac{C_{a}\left( t \right)}{C_{a}\left( t-7 \right)} \right]=\beta_{0}+\beta_{1}log\left( {NP}_{a}\left( t-L \right) \right)$ (S1)

The model used in both the Markov switching regression and time-varying regression is described in Eq. S1. Markov switching regression was conducted using the R package MSwM (1), and time-varying regression was conducted using the R package tvReg (2).

**Prior distributions that were used in the fixed effect linear regression analysis**

In the estimation of models described in Eqs. (5)–(9) in the main text, we used the normal distribution with a mean of 0 and standard deviation of 10. For $\sigma_{\omega}$, we used the standard half-Cauchy distribution, and for $\rho$, which is the autocorrelation coefficient for error $\varepsilon\left( t \right)$, we used the default prior of $uniform\left( -1, 1 \right)$.

**Supplementary Figure 1.** Aichi’s (A) daily reported COVID-19 case counts and (B) night-time population in designated areas from 10:00 PM to 11:59 PM are shown in the left column (light blue lines show 7-day moving averages). The results of Markov switching linear regressions assuming two hidden states (C) and time-varying linear regression (D) are shown in the right column. In all figures, light orange shading corresponds to four publicly declared “State of Emergency” periods.


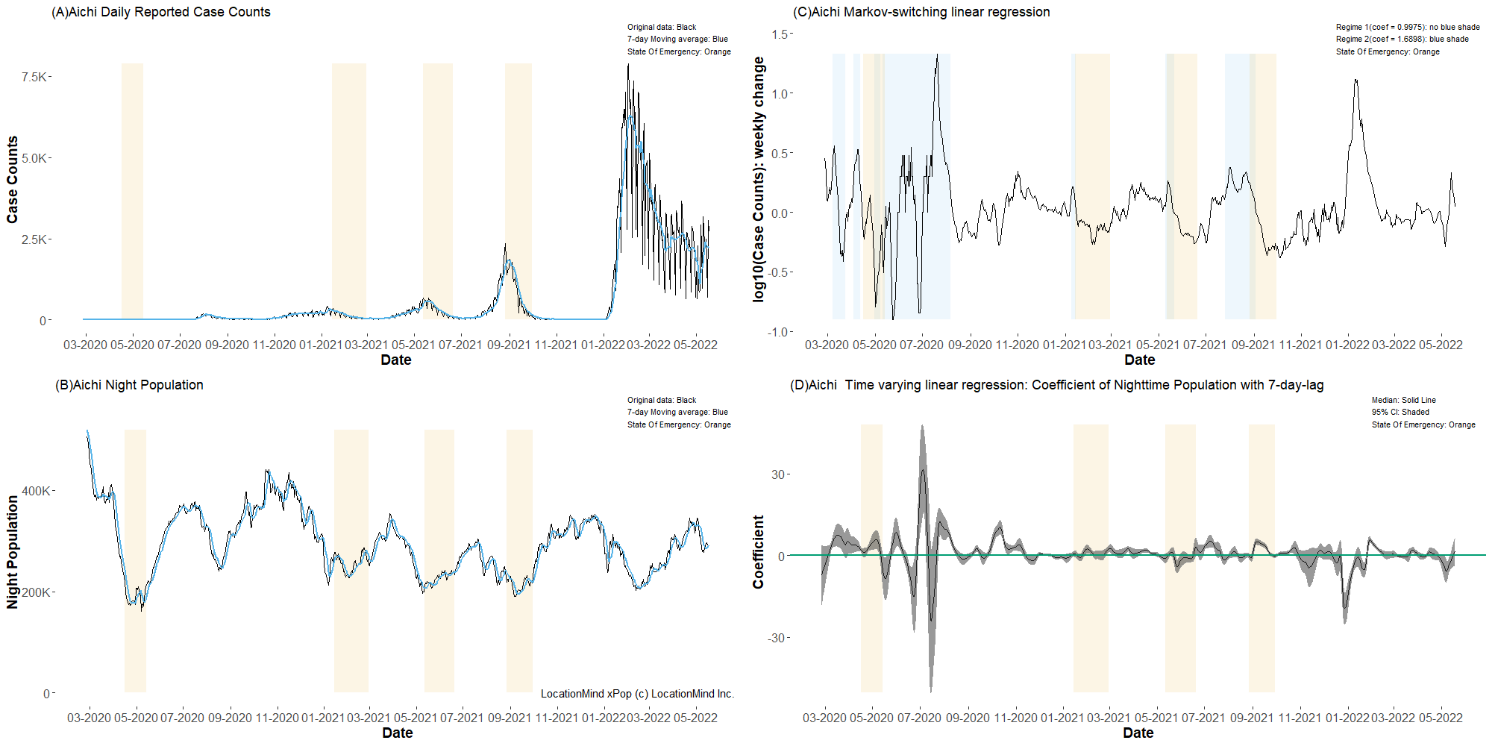


**Supplementary Figure 2.** Osaka’s (A) daily reported COVID-19 case counts and (B) night-time population in designated areas from 10:00 PM to 11:59 PM are shown in the left column (light blue lines show 7-day moving averages). The results of Markov switching linear regressions assuming two hidden states (C) and time-varying linear regression (D) are also shown in the right column. In all figures, light orange shading corresponds to four publicly declared “State of Emergency” periods.


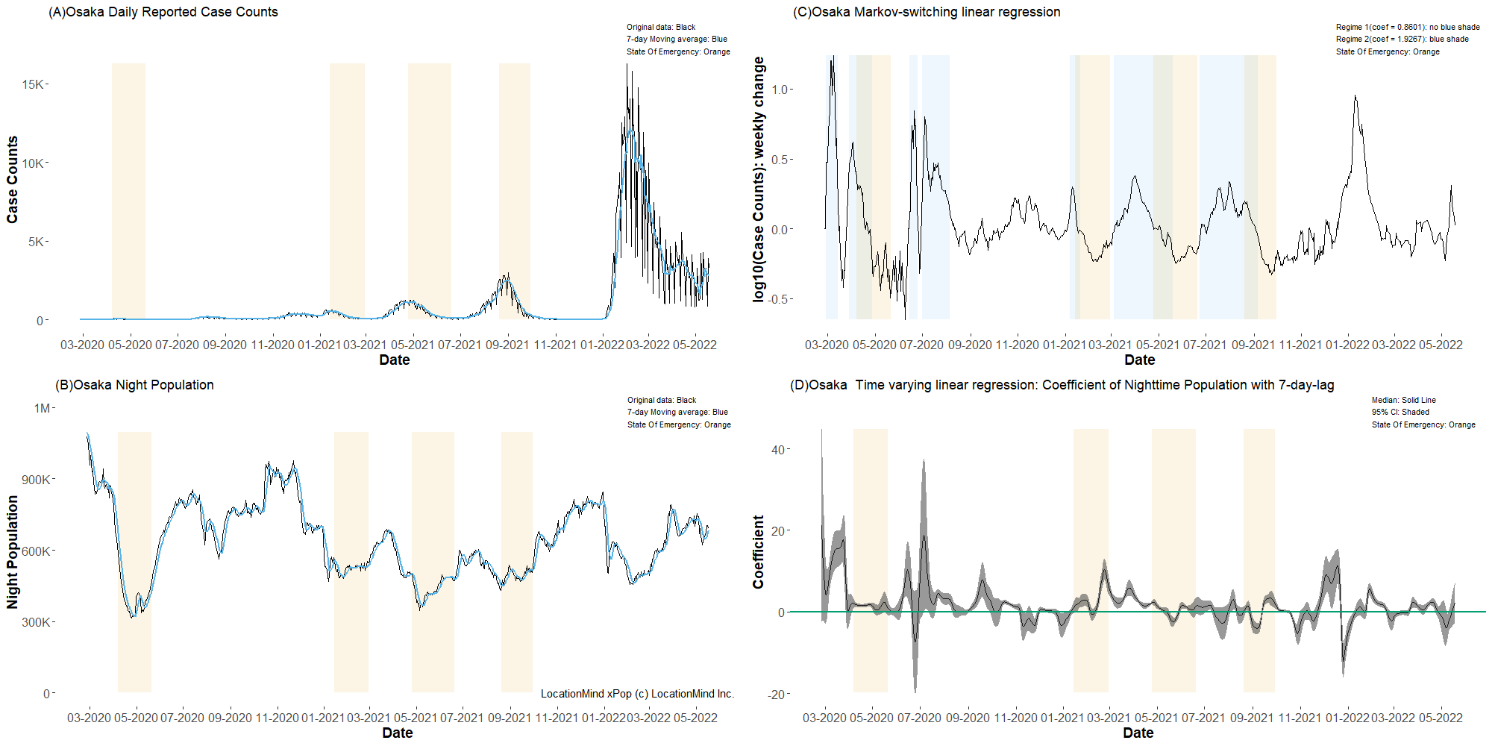


**Supplementary Figure 3.** Time series of residuals of the fixed-effect regression models for Tokyo, Aichi and Osaka.


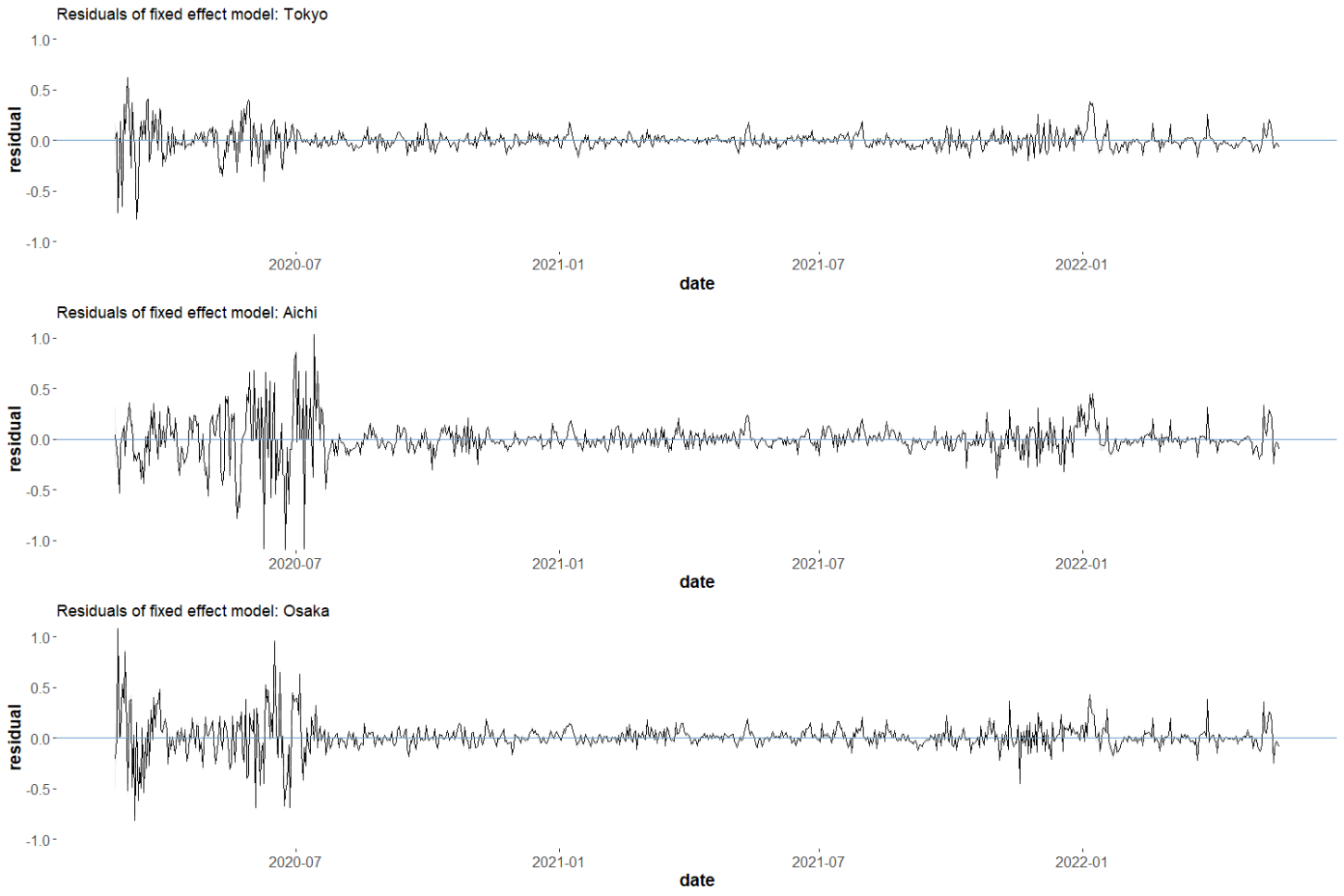


**Supplementary Figure 4.** Autocorrelation plots of residuals of the fixed-effect regression models for Tokyo, Aichi and Osaka.


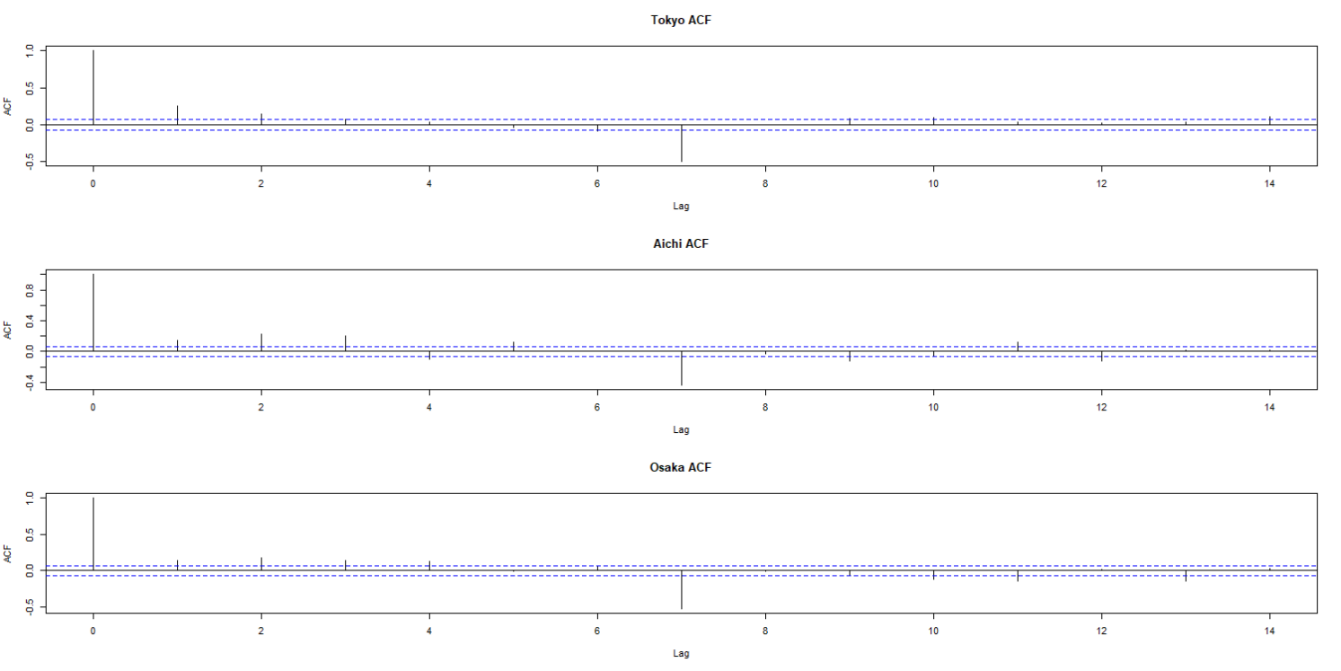


**Supplementary Figure 5.** Average lag between report to symptom onset in Tokyo Metropolitan calculated from the line list of anonymized COVID-19 cases reported as open data. Cases without information on symptom onset were excluded from our analysis. Dots are the lag average of all COVID-19 cases on each reporting date, and the blue line is the 7-day moving average.


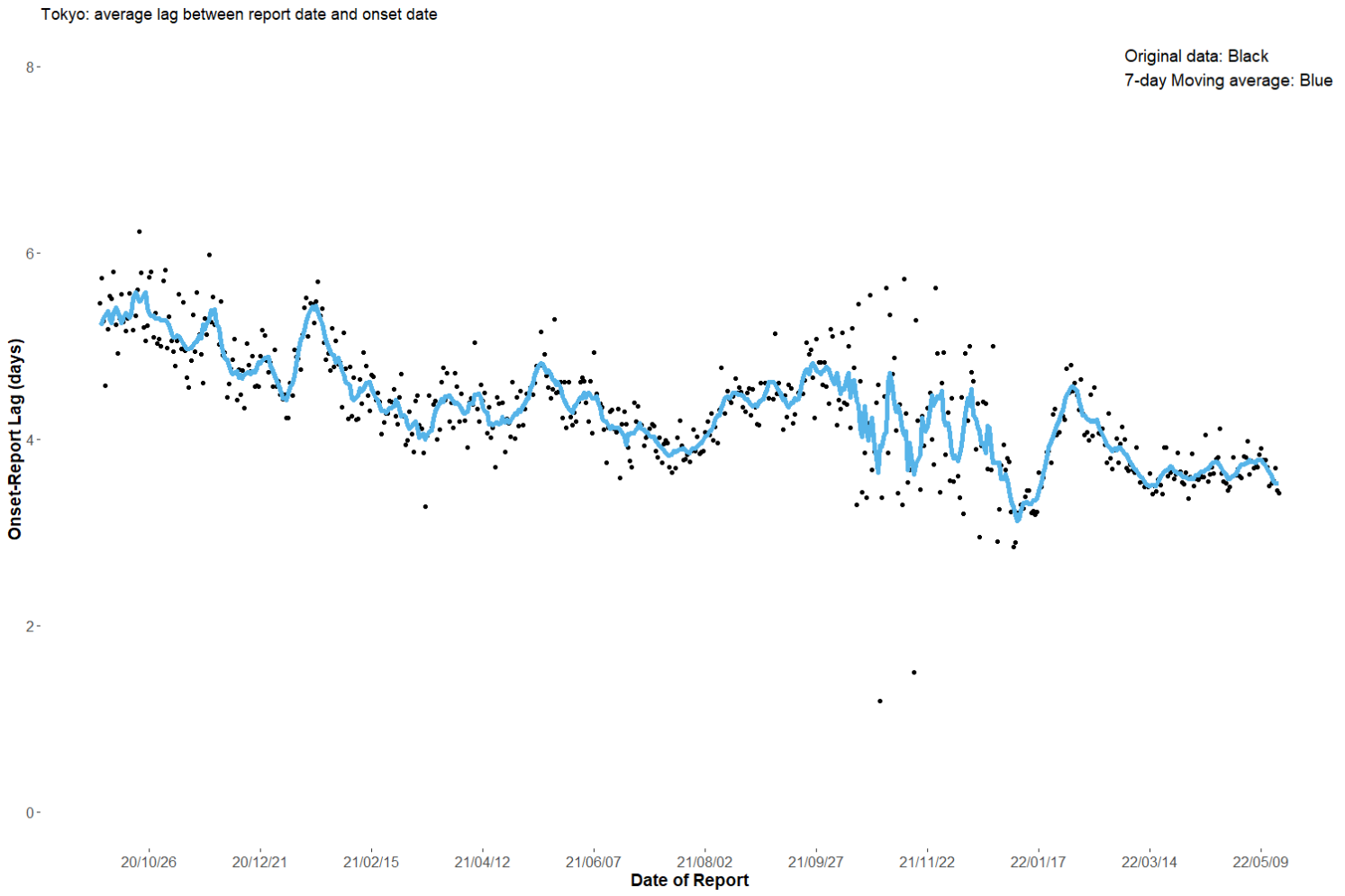


**Supplementary Table 1.** AIC values from the time-varying regression models for Tokyo, Aichi, and Osaka.

| lag | Tokyo | Aichi | Osaka |
| --- | --- | --- | --- |
| 7 days | -1657.88 | -548.265 | -934.549 |
| 8 days | -1691.05 | -555.054 | -1005.8 |
| 9 days | -1665.72 | -584.502 | -963.668 |
| 10 days | -1658.21 | -561.471 | -953.128 |
| 11 days | -1659.94 | -560.322 | -936.99 |
| 12 days | -1671.72 | -577.845 | -969.92 |
| 13 days | -1675.3 | -536.282 | -836.906 |
| 14 days | -1659.67 | -540.098 | -959.429 |

**Supplementary Table 2.** WAIC values from the fixed effect regression models for Tokyo, Aichi, and Osaka.

| Tokyo |  |  |  |  |
| --- | --- | --- | --- | --- |
|  | lag | log(NP) | log(NP)+Δlog(NP) | Δlog(NP) |
|  | 7 days | -1286.88 | -1297.345 | -1287.998 |
|  | 8 days | -1294.388 | -1328.647 | -1306.04 |
|  | 9 days | -1311.29 | -1309.541 | -1280.111 |
|  | 10 days | -1309.155 | -1308.836 | -1279.245 |
|  | 11 days | -1310.832 | -1308.594 | -1279.693 |
|  | 12 days | -1308.949 | -1309.498 | -1285.845 |
|  | 13 days | -1300.708 | -1298.843 | -1279.909 |
|  | 14 days | -1298.941 | -1299.719 | -1285.597 |
| Aichi |  |  |  |  |
|  | lag | log(NP) | log(NP)+Δlog(NP) | Δlog(NP) |
|  | 7 days | -500.172 | -500.662 | -478.759 |
|  | 8 days | -502.326 | -510.421 | -483.947 |
|  | 9 days | -510.617 | -509.277 | -478.464 |
|  | 10 days | -510.234 | -508.506 | -481.229 |
|  | 11 days | -505.62 | -503.188 | -478.947 |
|  | 12 days | -503.901 | -502.306 | -479.093 |
|  | 13 days | -502.941 | -503.789 | -484.598 |
|  | 14 days | -495.281 | -493.328 | -479.874 |
| Osaka |  |  |  |  |
|  | lag | log(NP) | log(NP)+Δlog(NP) | Δlog(NP) |
|  | 7 days | -737.359 | -737.404 | -731.254 |
|  | 8 days | -739.253 | -753.893 | -742.486 |
|  | 9 days | -749.596 | -748.815 | -735.021 |
|  | 10 days | -740.825 | -744.847 | -737.978 |
|  | 11 days | -736.379 | -735.449 | -732.14 |
|  | 12 days | -735.416 | -734.308 | -731.056 |
|  | 13 days | -736.05 | -742.001 | -739.325 |
|  | 14 days | -732.588 | -732.28 | -730.924 |
